# Strain Sharing Assessment in Woven Fiber Reinforced Concrete Beams Using Fiber Bragg Grating Sensors

**DOI:** 10.3390/s16101564

**Published:** 2016-09-22

**Authors:** Roberto Montanini, Antonino Recupero, Fabrizio De Domenico, Fabrizio Freni

**Affiliations:** Department of Engineering, University of Messina, Contrada di Dio, Messina I-98166, Italy; arecupero@unime.it (A.R.); ingfdd@aliceposta.it (F.D.D.); fabfreni@hotmail.com (F.F.)

**Keywords:** Fiber Bragg grating sensor, embedded optical fiber sensor, strengthened concrete structures, rehabilitation and retrofitting, strain measurements, fiber reinforced cementitious matrix (FRCM), strain transfer mechanism, 42.81.Pa, 81.05.Ni, 07.10.Pz

## Abstract

Embedded fiber Bragg grating sensors have been extensively used worldwide for health monitoring of smart structures. In civil engineering, they provide a powerful method for monitoring the performance of composite reinforcements used for concrete structure rehabilitation and retrofitting. This paper discusses the problem of investigating the strain transfer mechanism in composite strengthened concrete beams subjected to three-point bending tests. Fiber Bragg grating sensors were embedded both in the concrete tensioned surface and in the woven fiber reinforcement. It has been shown that, if interface decoupling occurs, strain in the concrete can be up to 3.8 times higher than that developed in the reinforcement. A zero friction slipping model was developed which fitted very well the experimental data.

## 1. Introduction

The conventional concrete rehabilitation technique by means of betòn-plaques has been proven to be an effective strengthening solution in civil engineering applications but it has many disadvantages such as high weight and corrosion due to exposure to harsh environments that may eventually increase the overall maintenance cost. This has encouraged research on new materials and technologies that allow for a reduction of the installation costs. The use of fiber reinforced plastics (FRPs) or fiber reinforced cementitious matrices (FRCMs) for concrete beam strengthening and column confinement is an example of the results of the research efforts devoted to this important task during the last few years and represents a noteworthy improvement with respect to traditional methods. In this context, deployment of embedded fiber optic sensors (FOSs) as a real-time health monitoring measurement system is very attractive, since it might provide a powerful method for substantially improving the durability and the safety of the retrofitted structure [1,2,3,4]. The chemical and mechanical compatibility of optical fiber sensors with concrete has been proven by several studies, showing that proper fiber coatings are able to withstand the highly alkaline chemical environment of concrete [5] as well as guarantee accurate strain transfer [6,7]. In addition, fiber Bragg grating (FBG) sensors have already been successfully embedded into FRP laminated composites to monitor thermal and residual strains in the host material [8,9]. 

Application of FOSs in concrete was first suggested by Mèndez et al. in 1989 [10], while the earliest use of FBG sensors as a structural health monitoring device in a real bridge structure was demonstrated some years later [11]. Since then, several research groups have reported on a variety of applications of FOSs embedded in concrete structures to detect strain, vibration, corrosion or crack development both in laboratory demonstrations [12,13,14,15,16,17] and field projects [18,19,20,21,22,23,24]. 

While the use of FOSs embedded in concrete has already been reported in several studies, their application for monitoring composite reinforcements used for concrete structures rehabilitation and retrofitting is much more recent. Sundaram et al. [25] and Wang et al. [26,27] investigated the mechanical behavior of woven reinforced concrete beams under four-point bending using embedded FBGs. The FBG strains were compared to those measured by externally bonded strain gauges, indicating good agreement between the load-strain curves in the linear elastic range, but it has to be noted that in these tests, externally bonded strain gauges were merely used for validating FBGs outputs.

In other two studies, Lau et al. [28] and Chan et al. [29] placed the FBGs at the interface between the concrete beam surface and the reinforcing composite patch (instead than embedding them into the composite patch), while still using externally bonded strain gauges for reference. They found that, in the linear elastic range, the strain at the interface was always higher than that measured at the tension-side reinforcement, although no explanation was furnished for this behavior. 

So far, the actual strain transfer mechanism between reinforcement and concrete was never evaluated. Indeed, this is thought to be a key factor for forecasting the incipient failure of the strengthened structure, since the knowledge of the interface strain alone might be insufficient in most cases and does not give information of whether the stress limit in the concrete or in the reinforcement is approaching. 

In this paper the problem of evaluating strain sharing in strengthened concrete beams has been addressed using FBG sensors embedded both into the concrete tensioned surface and into the woven fiber reinforcement. It has been shown that strain in concrete can be much higher than that developed in the reinforcement if relative slipping occurs. A theoretical model which is helpful for interpreting the obtained experimental results is also discussed.

## 2. Materials and Methods 

### 2.1. FBG Sensing Principle

In-fiber Bragg gratings exhibit dual sensitivity since their output relative wavelength shift (*Δλ_B_*/*λ_B_*) depends on either the applied strain variation or the temperature difference, as shown by the sensing equation [8]:

(1) $$\frac{\Delta \lambda_{B}}{\lambda_{B}}=(1-p_{e})\cdot \varepsilon +(\alpha_{f}+\xi )\cdot \Delta T=K_{\varepsilon }\cdot \Delta \varepsilon +K_{T}\cdot \Delta T$$

where *λ_B_* is the Bragg wavelength, *p*_e_ the photo-elastic constant of the optical fiber, *ξ* the thermo-optic constant, α_*f*_ the thermal expansion coefficient, Δ*T* the temperature interval (defined as the difference between the actual temperature *T* and the reference one *T*_ref_ at which *λ_B_* has been measured) and Δ*ε* the total strain variation along the grating due to the combined action of external forces and apparent strain induced by differential thermal expansion of the optical fiber relative to the host structure. These constants can be condensed into two coefficients, *K_ε_* and *K_T_*, which represent the strain sensitivity and the temperature sensitivity, respectively. If the two wavelength contributions of Equation (1) can be discriminated, it is then possible to measure simultaneously both temperature and strain.

### 2.2. Concrete Specimens Preparation

Three standard laboratory size rectangular concrete beams were prepared for three-point bending tests to be carry out according to the ASTM C293-94 standard. The concrete beams were manufactured using a wooden form with suitable liquid disarming. The final dimensions of the specimens are 400 × 85 × 85 mm (L × W × H). The concrete mixture used for casting was determined in relation to the desired class of strength (C 32/40 MPa), employing aggregates of three different sizes (up to 15 mm). Afterward 24 h, the beams were removed from the forms and cured in water at 20 °C for 28 days.

**Figure 1:**
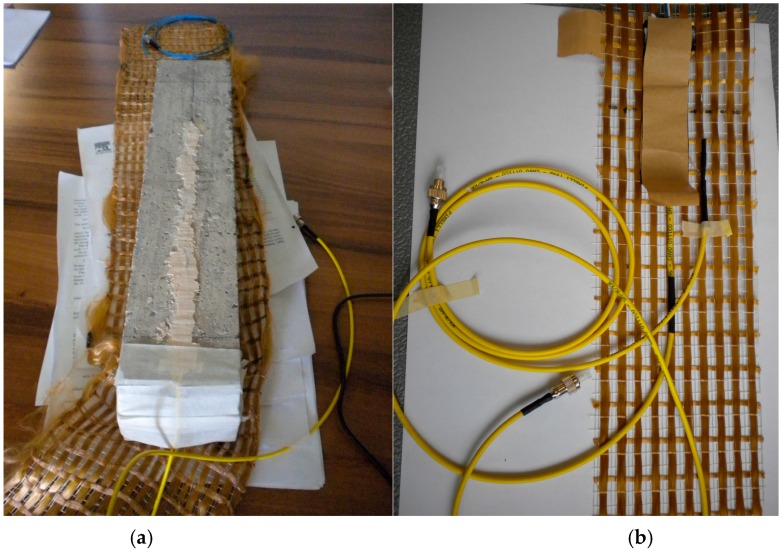
(**a**) Post-cured concrete beam with embedded FBG; (**b**) PBO reinforcing woven instrumented with FBGs and electrical strain gauge (before application).

After the aging, and before applying the reinforcement system, the beam was instrumented with a 1450 nm nominal wavelength FBG sensor (grating length: 20 mm; cladding diameter 125 μm, reflectivity >90%; reflective bandwidth @-3 dB < 0.3 nm; side lobe suppression >15 dB; cladding mode loss <0.2 dB; fiber SMF-28) glued with epoxy adhesive into a small groove that had been machined longitudinally into the concrete surface (see Figure 1a). The optical fiber (but not the grating) was fed in a yellow buffer to shield it and special care was devoted to protect the lead connecting to the laser source and interrogation system by using a small diameter plastic tube at the point where the fiber exits from the concrete. A second FBG sensor was also placed into the concrete groove to measure temperature. In this case, the grating has been encapsulated into a small diameter capillary [8], in order to isolate it from mechanical loads transmitted to the fiber when it is embedded into the concrete. To prevent loading of the encapsulated FBG caused by thermal expansion of the capillary material, the optical fiber has been cut out at one end by using an optical fiber cleaving tool and fixed at the other one by means of a high temperature resistant epoxy adhesive. This grating (FBG_T_) operates in a strain free condition and behaves as an optical fiber thermometer, allowing the measurement of the actual temperature, while the embedded grating (FBG_ε_) gives out a signal which depends on either temperature or strain.

### 2.3. Fiber Reinforced Cementitious Matrix Strengthening System

The strengthening system consisted of a fiber reinforced cementitious matrix (FRCM) with polyparaphenylene benzobisoxazole (PBO) bidirectional (0°/90°) mesh woven (Ruredil X Mesh Gold, S. Donato Milanese (Milan), Italy) [30]. It makes use of a monocomponent cementitious mortar that acts as the matrix, joining the high performance PBO fibers to the substrate of the concrete structure. In contrast to conventional carbon fiber reinforced plastics (CFRP), which use epoxy resin as binder, the PBO-FRCM system does not require any inter-phase (primer, resin, etc.), thereby considerably improving the structural performance of the reinforcement. 

**Figure 2:**
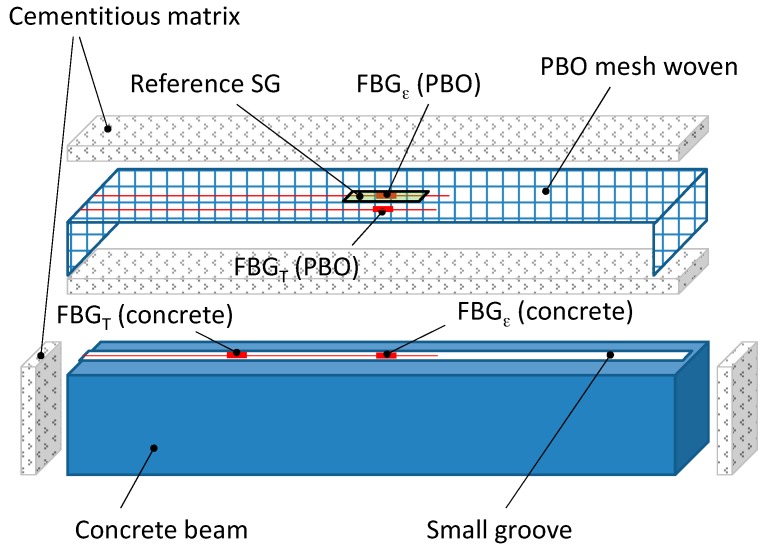
Schematic drawing of instrumented FRCM strengthened beam with embedded FBGs. (not in scale).

The PBO fabric was instrumented using two FBG sensors (grating length: 20 mm; cladding diameter 125 μm, reflectivity >90%; reflective bandwidth @-3 dB < 0.3 nm; side lobe suppression >15 dB; cladding mode loss <0.2 dB; fiber SMF-28) having different nominal wavelengths (1501 nm and 1547 nm), intended to measure the longitudinal strain and the temperature variation, respectively (Figure 1b). The latter FBG sensor was prepared in the same way as that used to measure temperature in concrete. A traditional electrical strain gauge (SG), having a grid length comparable to that of the Bragg gratings, was also attached onto the textile for reference. This SG was placed on the other side of the textile, opposite to FBG_ε_. A dummy gauge in half-bridge configuration was used in this case for temperature compensation. The instrumented patch was finally laid-up on the tensile and shear surfaces (i.e., U-shape round wrapping) of the concrete beam using two layers of cementitious mortar (total thickness of about 8 mm). Figure 2 shows a schematic of the experimental design.

### 2.4. Strain Measurements

Wavelength shifts from FBG sensors were measured by a multiwavelength-referenced FBG interrogation system (Micron Optics, Atlanta, GA, USA), which provides fiber optic sensors illumination by a broadband light-emitting diode (LED). The wavelength peak information is extracted from the reflected FBG spectrum through a single-fiber Fabry–Perot (FFP) tunable filter that scans the entire wavelength range; then the filtered output is electrically detected, differentiated and zero-crossing processed to achieve subpicometer wavelength measurement resolution. SG output was measured using a multi-channel acquisition system (Hottinger Baldwin Messtechnik GmbH, Darmstadt, Germany). Both FBG and SG values were recorded every second (1 Hz acquisition rate). 

### 2.5. Preliminary Experimental Tests on PBO Mesh Woven

**Figure 3:**
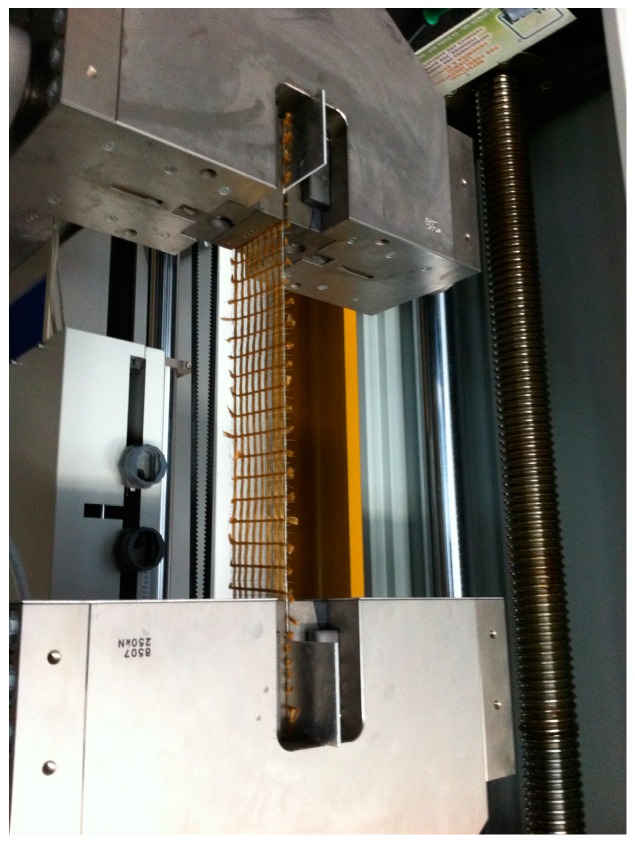
Tensile test carried out on the PBO mesh woven instrumented with FBG and strain gauge: Experimental set-up.

While embedding of FBG sensors into concrete has already been proved to be effective [1,2,3,4], measurement of the strain in PBO mesh woven by means of fiber optic sensors is more challenging, because of the very small diameters of the fibers of the reinforcement. To the authors’ knowledge, this was never done before. Therefore, in order to demonstrate the feasibility of the proposed approach, tensile tests were preliminary carried out on the PBO mesh woven instrumented with FBG sensors, using strain gauge for reference purpose (Figure 3). A quasi-static universal testing machine (Allround Z250, Zwick/Roell, Ulm, Germany) was employed to carry out these tests. The mesh woven was carefully aligned to the frame and fixed by means of wedge-screw grips. Tests were performed in force control with 100 N preload and 10 N·s^−1^ cross-head speed. 

### 2.6. Three Point Bending Tests on Woven Fiber Reinforced Concrete Beams

**Figure 4:**
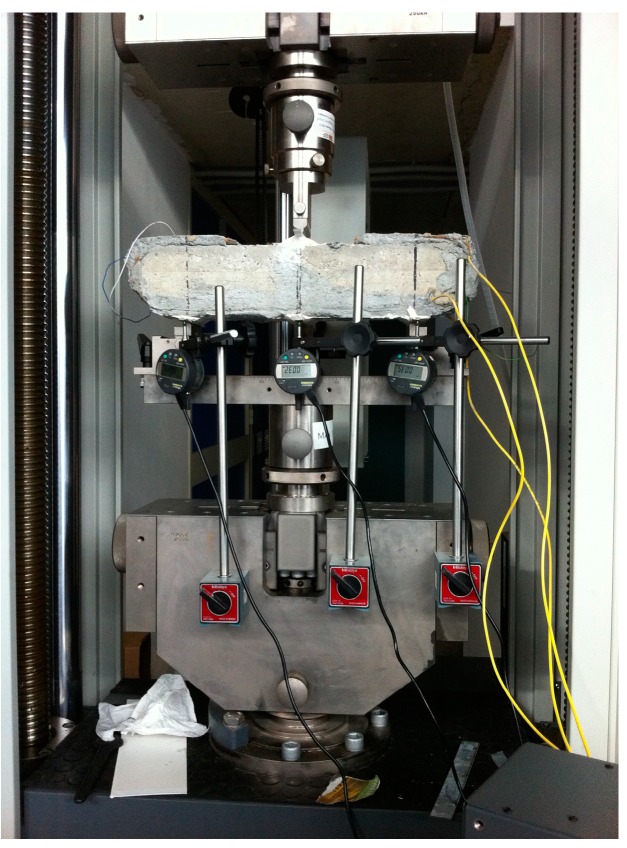
Three-point bending test carried out on FRCM strengthened beam instrumented with FBGs: Experimental set-up.

Three-point bending tests were carried out by mounting the composite-reinforced concrete beams with 300 mm span test fixtures (Figure 4). Three mechanical comparators were used for alignment purposes. Tests were performed under force control with 200 N preload and 10 N·s^−1^ cross-head speed. Three tests were performed to assess repeatability.

## 3. Theoretical Model

As already explained in Section 2.3, the FRCM strengthening system applied to the bottom surface of the concrete beam consists of a synthetic polymeric textile embedded into a cementitious matrix. In this section, a theoretical model is developed which is able to reproduce the flexural behavior of the strengthened beam. The following two hypotheses have been assumed:

i) The whole cross-section of the concrete beam (*B* × *h_c_*) is able to withstand the applied load until failure occurs;
ii) The tensile strength of the cementitious matrix is negligible with respect to that of the PBO.

**Figure 5:**
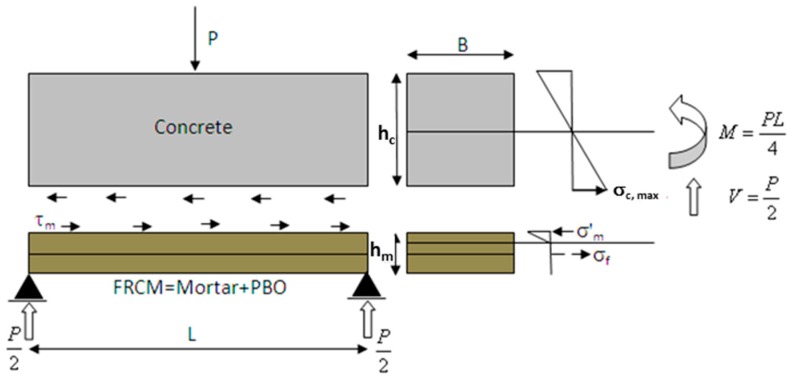
Theoretical model: zero friction slipping hypothesis.

Then, the concrete beam and the FRCM could be considered as two de-coupled systems, which exchange shear stresses through the bonded interface (Figure 5). 

If the two curvatures are set equal, the bending moments can be written as:

(2) $$\begin{array}{ccc} \left\{ \begin{array}{l} \frac{M_{c}}{E_{c}I_{c}}=\frac{M_{r}}{E_{m}I_{r}}\\ M=M_{c}+M_{r} \end{array}\right. & \text{and} & \left\{ \begin{array}{l} M_{r}=\left(\frac{E_{m}I_{r}}{E_{c}I_{c}+E_{m}I_{r}}\right)M\\ M_{c}=M-M_{r} \end{array}\right. \end{array}$$

where the subscripts “*c*”, “*r*” and “*m*” refer to the concrete beam, the FRCM reinforcement system and the cementitious matrix, respectively. In the same way, if the angular distortions are set equal, the shear force in concrete and in mortar can be can be written as:

(3) $$\begin{array}{ccc} \left\{ \begin{array}{l} \frac{V_{c}}{G_{c}A_{c}}=\frac{V_{r}}{G_{m}A_{r}}\\ V=V_{c}+V_{r} \end{array}\right. & \text{and} & \left\{ \begin{array}{l} V_{r}=\left(\frac{G_{m}A_{r}}{G_{c}A_{c}+G_{m}A_{r}}\right)V\\ V_{c}=V-V_{r} \end{array}\right. \end{array}$$

where *G*_c_ and *G*_m_ represent the shear modulus of concrete and mortar, respectively, which can be expressed as a function of Young’s modulus and Poisson’s ratio:

(4) $$G_{c}=\frac{E_{c}}{2(1+\nu_{c})},\;G_{m}=\frac{E_{m}}{2(1+\nu_{m})}$$

The maximum tensile stress reached in the cross-section as the beam is loaded with a bending moment *M*_c_ is:

(5) $$\sigma_{c,\text{max}}=\frac{M_{c}}{I_{c}}\frac{h_{c}}{2}$$

where:

(6) $$I_{c}=\frac{B{h_{c}}^{3}}{12}$$

while the maximum compressive stress in the mortar cross-section is:

(7) $${\sigma^{\prime }}_{m}=\frac{M_{r}}{I_{r}}x_{m}$$

where:

(8) $$x_{m}=-\frac{n_{mf}A_{f}}{B}+\left[-1+\sqrt{1+\frac{2BA_{f}\left(\frac{h_{m}}{2}\right)}{n_{mf}{A_{f}}^{2}}}\right]$$

represents the distance from the top fibers to the neutral axis and depends on the PBO textile cross-section *A_f_* = *B* × *h_f_* (in which the subscript “*f*” has been used to indicate the fibers of the PBO textile) and on the homogenization coefficient of the mortar relative to the reinforcing fiber:

(9) $$n_{mf}=\frac{E_{f}}{E_{m}}$$

By writing the moment of inertia *I_r_* as:

(10) $$I_{r}=\frac{B{x_{m}}^{3}}{3}+n_{mf}A_{f}{\left(\frac{h_{m}}{2}-x_{m}\right)}^{2}$$

The tensile stress into the PBO fiber can be calculated as:

(11) $$\sigma_{f}=n_{mf}\frac{M_{r}}{I_{r}}\left(\frac{h_{m}}{2}-x_{m}\right)$$

Finally, the average shear stress τ_*m*_ acting through the concrete-mortar interface can be computed as:

(12) $$\tau_{m}=\gamma_{m}G_{m}$$

where:

(13) $$\gamma_{m}=\chi \frac{V_{r}}{G_{m}A_{r}}$$

is evaluated in terms of the shear factor *χ* (*χ* = 6/5 for a rectangular cross section) and the homogenized section of the reinforcement *A_r_*, defined as:

(14) $$A_{r}=A_{m}+n_{mf}A_{f}$$

Therefore, the stresses at the concrete-mortar (*σ_cm_*) interface and at the mortar-PBO textile (*σ_fm_*) interface can be obtained as:

(15) $$\sigma_{cm}=\sigma_{c,\text{max}}-{\sigma^{\prime }}_{m}-n_{cm}\tau_{m}$$

(16) $$\sigma_{fm}=\sigma_{f}+n_{fm}\tau_{m}$$

with:

(17) $$n_{cm}=\frac{E_{m}}{E_{c}}$$

Hence, the strains in concrete and in the FRCM reinforcement are finally obtained as:

(18) $$\varepsilon_{c}=\frac{\sigma_{cm}}{E_{c}}$$

(19) $$\varepsilon_{FRCM}=\frac{\sigma_{fm}}{E_{f}}$$

Using Equations (18) and (19), the strain components can be evaluated in any section of the beam as a function of the applied external load. Table 1 reports nominal data used for computing predicted strains in in the concrete beam and in the FRCM reinforcement.

## 4. Results and Discussion

**Figure 6:**
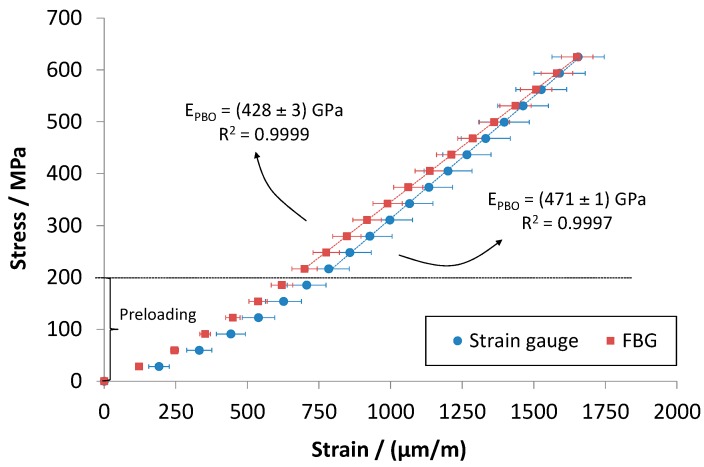
Tensile tests carried out on PBO textile instrumented with FBG and strain gauge: Averaged stress-strain curves.

Figure 6 reports average stress-strain curves obtained after a tensile test on the PBO woven mesh. This test served to validate FBG strain measurements, proving that it is possible to measure accurately the PBO elongation by means of a FBG bonded on it. Stresses were computed by measuring the actual width of the textile using the net pitch data provided by the manufacturer. 

**Figure 7:**
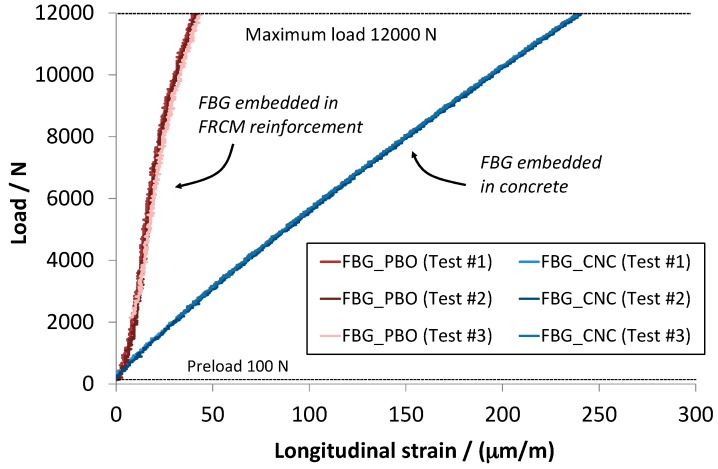
Three-point bending tests carried out on FRCM-reinforced concrete beams with embedded FBGs: Load-strain curves measured in concrete and in FRCM reinforcement.

The obtained results showed that FBG strain values are in good agreement with those measured by the bonded electric strain gauge, since the observed difference is not statistically significant. Moreover, by interpolating stress-strain curves in the range between 200 MPa and 600 MPa, the Young modulus of the PBO can be estimated. Whether FBG or SG data are used, a value of 428 ± 3 GPa or 471 ± 1 GPa has been obtained for E_PBO_. Note the both values are close to that stated by the manufacturer (450 GPa, see Table 1), resulting in a deviation of −4.9% and +4.7%, respectively. Figure 7 reports experimental load-strain curves for concrete and FRCM reinforcement after three-point bending tests.

**Figure 8:**
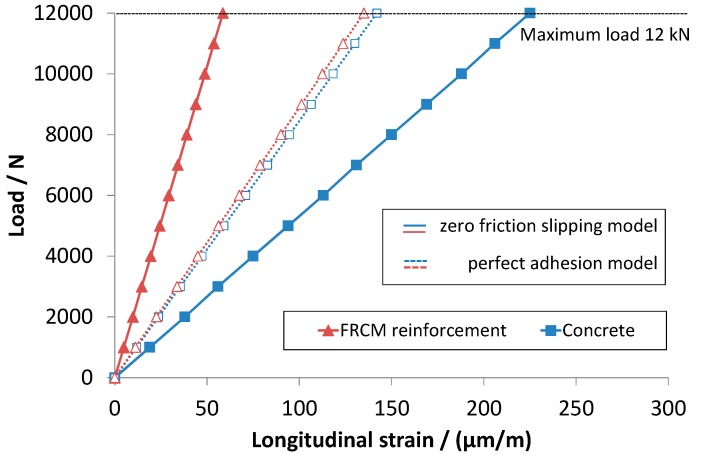
Load-strain curves obtained by applying Equations (18) (concrete) and (19) (Fiber Reinforced Cementitious Matrix) with data reported in Table 1.

The maximum load reached was relatively lower than the ultimate load of the concrete, which is about 14 kN. The reinforced beams worked in the linear elastic range, and even after repeated tests, no cracks or visible failures appeared on any of them. However, the load-strain curves present some degree of non-linearity. At low loads, non-linearity has to be attributed to local effects produced at the interface between the machine loading frames and the specimen (note that, in order to avoid excessive load concentration, small steel plates were used at the supporting and loading sites). As load is increased, non-linearity can no longer be associated to beam loading/supporting adjustments. More importantly, results of bending tests underlined a marked difference between the strain values measured in the concrete tensioned surface and those recorded in the reinforcement, highlighting inefficient load transfer. This behavior may be explained by predicting a relative slipping between the concrete and the cementitious matrix. By assuming zero friction between the two materials, a complete decoupling will occur and the deflections of the two beams become independent from each other. Therefore, the bending moments into the two virtual beams are proportional to the respective bending stiffness. The observed experimental trends can be predicted by using the zero friction slipping model developed in Section 3. In fact, application of Equations (18) and (19) with data reported in Table 1 leads to the load-strain curves of Figure 8, which display a close agreement with the experimental ones. According to the theoretical model, at 12 kN load, the expected strains on the tensioned surface of the concrete beam and on the FRCM reinforcement are 225 µm/m and 58.6 µm/m, respectively. If perfect adhesion (no slipping) is assumed instead, strain values of 142 µm/m and 135 µm/m should be attained (see Figure 8). Since the measured strains are 240 ± 5 µm/m and 40 ± 4 µm/m, it can be concluded that, for the FRCM concrete beam, an almost complete decoupling was actually achieved in our experiments.

The relative slipping between the concrete and the PBO mesh woven is mainly due to the peculiar characteristics of the mortar used as matrix of the FRCM system. Although the bond of a multi-filament yarn in a fine grained cementitious matrix is obviously controlled by the bond properties between filaments and matrix, more detailed information is needed to evaluate the mechanisms of such a complex system under a pull-out load. Recent pull-out tests conducted by Banholzer et al. [7] have brought to light that the penetration of the mortar between the yarns of a mesh woven does not occur homogeneously. In fact, unlike epoxy resins used in conventional FRP, the mortar used in FRCM has a grained composition that can hardly sink evenly in all the spaces between the filaments of woven. However, the exactly amount of slipping is difficult to be predicted, since the application of the cement matrix is a hand-made process and its diffusion into the textile depends on a number of variables (e.g., temperature, cement granulometry, humidity, etc.). In this framework, the use of embedded FBG sensors might provide invaluable information that could not be obtained using externally bonded strain sensors.

## 5. Conclusions

This paper discussed the problem of evaluating strain sharing in woven fiber strengthened concrete beams subjected to three-points bending in the linear elastic range. Fiber Bragg grating sensors were embedded both in the concrete and in the reinforcement. A theoretical model was also developed for interpreting the obtained experimental outcomes. The main findings can be summarized as follows:

1) Strain in concrete can be much higher than that developed in the reinforcement if slipping between the interface surfaces occurs.
2) For FRCM strengthened concrete beams, almost complete de-coupling has been observed. A zero friction slipping model was developed which fit very well the experimental data.
3) Otherwise, if perfect adhesion occurs, strains in concrete and in reinforcement tend to be similar.

## Figures and Tables

**Table 1:** Nominal data used for computing strain values in the concrete beam and in the FRCM reinforcement.

Symbol	Value	Unit	Description
L	300	mm	distance between supports
B	85	mm	beam cross section
h_c_	85	mm	concrete cross section height
h_m_	8	mm	mortar cross section height
h_f_	0.05	mm	PBO cross section height
E_c_	39,300	MPa	concrete modulus of elasticity
E_m_	6200	MPa	mortar modulus of elasticity
E_f_	450,000	MPa	PBO modulus of elasticity
ν_c_, ν_m_	0.25	-	Poisson’s number
